# Gut Microbiota Alteration Is Associated With Cognitive Deficits in Genetically Diabetic (Db/db) Mice During Aging

**DOI:** 10.3389/fnagi.2021.815562

**Published:** 2022-01-26

**Authors:** Jiawei Zhang, Yaxuan Zhang, Yuan Yuan, Lan Liu, Yuwu Zhao, Xiuzhe Wang

**Affiliations:** Department of Neurology, Shanghai Jiao Tong University Affiliated Sixth People’s Hospital, Shanghai, China

**Keywords:** type 2 diabetes, cognitive dysfunction, gut microbiota, neuroinflammation, aging

## Abstract

Recent studies have revealed that the microbiota may be implicated in diabetes-related cognitive dysfunction. However, the relationship between gut microbiota and cognitive dysfunction during the progression of type 2 diabetes remains elusive. We used 16S rRNA sequencing combined with conventional behavioral tests to explore the longitudinal changes of gut microbiota and cognition in diabetic db/db mice (leptin receptor knockout mice) and their wild-type littermates at different ages. Prussian blue staining was performed to detect the microhemorrhage in the brain, and immunofluorescent study was applied to analyze microglia activation. Moreover, a Meso Scale Discovery kit was used to determine the cytokine levels in the brain. Db/db mice exhibited age dependent pathological characteristics, including cognitive deficits, neuron damage, spontaneous hemorrhages and neuroinflammation. Furthermore, we observed that the diversity and composition of gut microbiota significantly differed between the wild-type and db/db mice during aging. We found that compared to age-matched wild-type mice, genus Helicobacter was significant higher in db/db mice at 18 and 26 weeks. Correlation analysis revealed that Helicobacter is positively associated with Iba-1 positive cells and TNF-α expression. Collectively, our longitudinal study suggests that diabetic cognitive impairment during aging is associated with abnormal gut microbiota composition, which may play a role in the regulation of neuroinflammation.

## Introduction

Type 2 diabetes (T2DM) is a chronic metabolic disorder and its prevalence is on the rise throughout the world. It causes serious complications in various organs, including the central nervous system (Zhao et al., 2019). Numerous studies have reported that diabetic patients have a high risk of developing cognitive impairment which is also termed as “diabetes-related cognitive dysfunction” (Umegaki, 2018; Liu et al., 2020; van Sloten et al., 2020). Diabetes-related cognitive dysfunction is manifested as decreased learning and memory, weakened executive ability, and emotional disturbance. Considering the prevalence of T2DM, diabetes-related cognitive dysfunction will inevitably bring arduous challenges and an immeasurable economic burden to social public health. Nevertheless, the knowledge on diabetes-related cognitive dysfunction is still insufficient, and available treatments for this complication are currently not effective enough. Therefore, it is imperative to explore the underlying pathogenesis of cognitive dysfunction caused by diabetes, and new strategies to delay or reduce the occurrence of the disease are needed.

As a metabolic disease, T2DM coexists with chronic inflammation, which has been considered as a key factor that contributed to the development of neurodegenerative diseases (Sankar et al., 2020). Studies have shown that neuroinflammation caused by persistent over-activation of microglia is implicated in the pathophysiology of cognitive impairment (Jackson et al., 2020). Activated microglia can release a variety of neurotoxic pro-inflammatory factors including Interleukin 6 (IL-6) and tumor necrosis factor α (TNF-α), which further activates microglia to aggravate neuroinflammation. The etiology of diabetes-related cognitive dysfunction may be multi-factorial. Recent studies have revealed that patients with T2DM and control subjects differed in gut microbiota composition (Karlsson et al., 2013; Bakir-Gungor et al., 2021). Microbiota homeostasis is not only essential for the maintenance of gut health, but also affects central nervous system by regulating the release of neurotransmitters and inflammatory factors (Collins et al., 2012). A number of studies have shown that gut microbiota composition and diversity of Alzheimer’s disease patients were different from those of healthy controls (Cattaneo et al., 2017; Sun et al., 2020). However, there are no relevant reports on how the gut microbiota changes with the progression of T2DM and whether it is related to diabetic cognitive dysfunction.

Given the potential role of gut microbiota in diabetes-related cognitive dysfunction, we undertook a comprehensive study to investigate the longitudinal changes of gut microbiota in db/db mice, a typical T2DM rodent model, using 16S ribosomal RNA sequencing combined with conventional behavioral tests and pathological analysis.

## Materials and Methods

### Animals

Male db/db (BKS-Lepr^*em*2C*d*479^/Gpt) and age-matched WT (wild-type) (C57BLKS/JGpt) mice were purchased from Jiangsu GemPharmatech Biotechnology Co., Ltd. (Jiangsu, China) and housed in a specific pathogen-free animal center under controlled temperature (20–25°C) and light (12 h light/12 h dark) conditions, with water and food available *ad libitum* until 6, 18, and 26 weeks of age. Body weight and fasting blood glucose level was measured at the end of the experiment. All procedures were performed in accordance with the principles outlined in the National Institutes of Health (NIH) Guide for the Care and Use of Laboratory Animals. The study was approved by the ethical committee on animal welfare of Shanghai Jiao Tong University Affiliated Sixth People’s Hospital.

### Morris Water Maze Test

The Morris Water Maze (MWM) test was performed to measure the spatial learning and memory as we described previously (Zhang J. et al., 2021). The utilized maze consisted of a circular pool (height 50 cm, diameter 120 cm), divided into four quadrants, filled up to a depth of 30 cm with tepid water (25 ± 1°C). A submerged escape platform (10 cm in diameter) 1 cm below the water surface was used for training. The maze was surrounded by curtains with visual cues of four different shapes and sizes placed in the four quadrants. Hidden platform task consisted of four trials per day on 5 consecutive days. Mice were allowed to swim for a maximum trial duration of 60 s and with 10 s on the platform at the end of the trials. During each trial, the latency required to reach the platform was measured as the escape latency. The platform was withdrawn at the sixth day of training for probe trial. The mice were released from the 4th quadrant which is opposite to the target quadrant and allowed to navigate freely for 60 s. During the probe trial, the number of times across the retracted platform, the percentage of time spent in the target quadrant and the average swimming speed were recorded.

### Novel Object Recognition Test

The NOR test was performed as described previously with slight modifications (Wang et al., 2020). In brief, on the first day, mice were habituated to experimental apparatus (40 cm × 40 cm × 50 cm) in the absence of objects for 5 min. On the second day, in the training phase, mice were exposed to two identical cubes which were fixed 9 cm from the wall for 5 min. A short-term memory test was performed 1 h later, mice were allowed to explore the apparatus for 5 min in the presence of the familiar cube and the novel triangular object. On the third day, to examine long-term memory, mice were allowed to explore the apparatus for 5 min in the presence of the familiar cube and the novel cylinder. In each phase, the amount of time mice spent exploring each object was recorded and the discrimination index was calculated as [(time with novel object − time with familiar object)/(time with novel object + time with familiar object)].

### Nissl Staining

After behavioral experiments, mice were transcardially perfused with 0.9% saline followed by 4% paraformaldehyde. And then the brains were embedding in paraffin and sectioned into 8-μm thick slices by a microtome for further staining. Briefly, the brain sections were deparaffinized, gradually rehydrated in graded concentrations of ethanol, and treated with conventional Nissl staining solution and the images were obtained using an optical microscope (× 100 and × 400).

### Prussian Blue Staining

The Prussian blue staining followed the procedures described previously (Villarreal et al., 2017). In short, slides were washed in distilled water and then immersed in a working solution of Prussian blue (5% hydrochloric acid and 5% potassium ferrocyanide). Slides were rinsed with distilled water and then counterstained for 5 min using Nuclear Fast Red. A final series of distilled water washes were performed before dehydration and being coverslipped. Then, the Prussian blue staining images were obtained using an optical microscope (× 40 and × 400).

### Immunofluorescence Staining

Paraffin sections were deparaffinized, rehydrated, and subjected to heat-induced antigen retrieval using a microwave, then rinsed in distilled water. The sections were blocked in 3% bovine serum albumin (BSA, Servicebio) for 30 min, and then incubated in anti-Iba-1 primary antibody (1:500, mouse, servicebio) at 4^°^C overnight. On the second day, the sections were then rinsed in 0.01-M PBS (pH 7.4) and incubated with an anti-mouse HRP-conjugated secondary antibody for 1 h at room temperature. Finally, Immunoreactivity was visualized using diaminobenzidine tetrahydrochloride (DAB), and the sections were stained with hematoxylin and mounted. The localization and distribution of immunoreactive positive cells in the brain were observed using a microscope (× 40 and × 400, IX53, Olympus, Tokyo, Japan).

### Meso Scale Discovery for Inflammatory Cytokines

The Meso Scale Discovery kit (MSD, Meso Scale Diagnostics, Rockville, MD, United States) was used for cytokine detection following the manufacturer’s instructions. Briefly, the whole brain was lysed and protein supernatants were quantified using a BCA kit. Fifty microliter of sample and standard were added in antibody-coated 96-well plates for incubation followed by washing three times with PBST. And then 25 μl of the prepared detection antibody was added to each well and the plates were sealed with parafilm and shaked at room temperature for 2 h. After washing three times with PBST, 150 μl of reading buffer was added in the plates. Cytokine levels were determined using a MESOTM QuickPlex SQ 120 (Meso Scale Diagnostics, Rockville, MD, United States).

### 16S rRNA Gene Sequencing of Fecal Samples

DNA from fecal samples was extracted using the E.Z.N.A.^®^ Stool DNA Kit (D4015, Omega, Inc., United States) according to manufacturer’s instructions. The V3-V4 region of the prokaryotic (bacterial and archaeal) small-subunit (16S) rRNA gene was amplified with universal primers 341F and 805R. The 5′ ends of the primers were tagged with specific barcodes per sample and sequencing universal primers. The PCR products were purified by AMPure XT beads (Beckman Coulter Genomics, Danvers, MA, United States) and quantified by Qubit (Invitrogen, United States). The amplicon pools were prepared for sequencing and the size and quantity of the amplicon library were assessed on Agilent 2100 Bioanalyzer (Agilent, United States) and with the Library Quantification Kit for Illumina (Kapa Biosciences, Woburn, MA, United States), respectively. The libraries were sequenced on NovaSeq PE250 platform.

### Statistical Analysis

Statistical analysis was performed using GraphPad Prism 7 (GraphPad Software Inc., La Jolla, CA). The data were presented as mean ± standard error of the mean (SEM). Differences between groups were performed using one-way analysis of variance (ANOVA) followed by a false discovery rate (FDR) correction for multiple comparisons. Correlation analysis was conducted using Spearman rank correlation analysis. For the hidden-platform training of the Morris water maze test, the escape latency was analyzed by two-way repeated-measures ANOVA followed by Tukey’s *post hoc* test. Statistical significance was set at *p* < 0.05.

## Results

### Learning and Memory Performance Was Impaired in Db/db Mice

**FIGURE 1:**
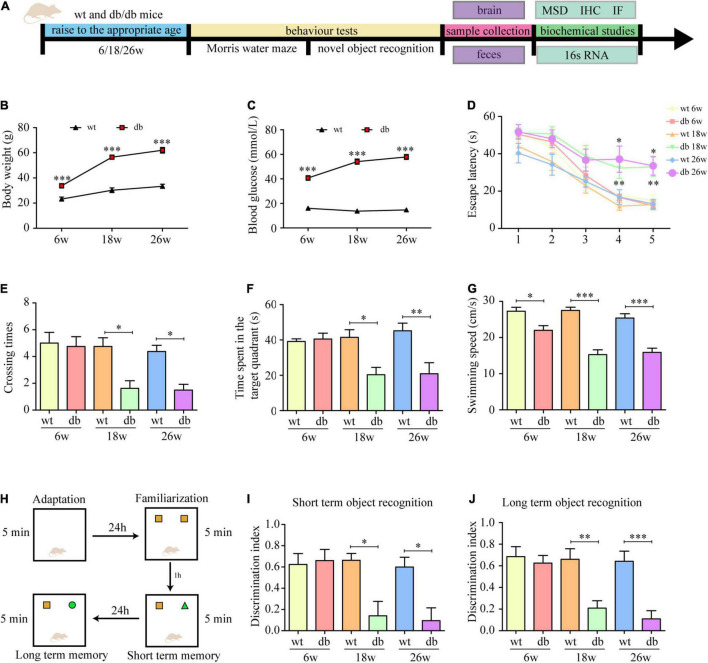
Learning and memory performance was impaired in db/db mice. **(A)** Schematic diagram of the experimental procedure. **(B)** Body weight at different ages. **(C)** Blood glucose at different ages. **(D)** The escape latency of mice in the training trials of the hidden platform task. **(E)** Frequency of platform crossing in the probe trial. **(F)** Percentage of time spent in the target quadrant in the probe trial. **(G)** Swimming speed in the probe trial. **(H)** The experimental design of novel object recognition test. **(I)** The discrimination index of 1 h test trials. **(J)** The discrimination index of 24 h test trials. (*n* = 8 per group); *P < 0.05; ^**^P < 0.01; ^***^P < 0.001.

Consistent with previous studies, the db/db mice in our study developed T2DM with age. Compared with age-matched WT mice, db/db mice had a phenotype of increased blood glucose and weight gain at 6 weeks, but the phenotype became more obvious as time reached 18 weeks or even 26 weeks (Figures 1A–C). To determine the effect of T2DM and age on cognitive function, spatial learning and memory performance was assessed via Morris water maze in 6, 18, and 26 weeks db/db and WT mice. The results revealed that the latency to reach the platform of WT mice was not statistically different acrossing the age, as indicated by a non-significant difference in the latency from 1st to 5th days at the age of 6, 18, and 26 weeks (Figure 1D). Db/db mice showed an age-dependent decline in the learning capacity indicated by a significant increase in the escape latency on the 4th and 5th days at the age of 18 and 26 weeks in comparison to 6 weeks (Figure 1D). For the factor T2DM, no significant difference was observed in 6-week-old WT and db/db mice. However, a significant effect of T2DM was observed at the age of 18 weeks, with db/db mice consistently taking longer time to reach the platform than WT mice, especially on the 4th and 5th days. A similar performance was observed in 26-week-old db/db mice as compared to age-matched WT mice (Figure 1D). To evaluate memory preservation, mice were subjected to a probe trial 24 h after the last training session. The number of times across the retracted platform and the percentage of time spent in the target quadrant were taken as an index of mice’s memory capacity. The analysis revealed that 18- and 26-week-old db/db mice showed difficulties remembering where the platform was originally placed and spent significantly shorter time in the target quadrant when compared to the rest of the groups (Figures 1E,F), supporting the cognitive impairment observed during the training phase.

We also routinely assessed the motor function of the mice and found that the swimming speed of the db/db mice at 18 and 26 weeks was significantly slower than that of the other groups (Figure 1G). To ensure that the cognitive dysfunction observed in the MWM were not attributed to the limitation in locomotor activity, NOR, a test less affected by motor ability, was used to further evaluate the cognitive impairment in mice. The analysis revealed that the discrimination index of WT and db/db mice at the age of 6 weeks was no statistically significant difference. At the age of 18 and 26 weeks, WT mice performed normally as shown by a significantly higher discrimination index for the novel object compared to the familiar object, while no significant difference discrimination index was found for the age-matched db/db mice (Figures 1H–J), indicating memory impairment of these mice. Collectively, the results in the behavioral tests indicate that learning and memory performance is impaired during the progression of T2DM in db/db mice.

### Brain Pathology Was Severe in Db/db Mice

**FIGURE 2:**
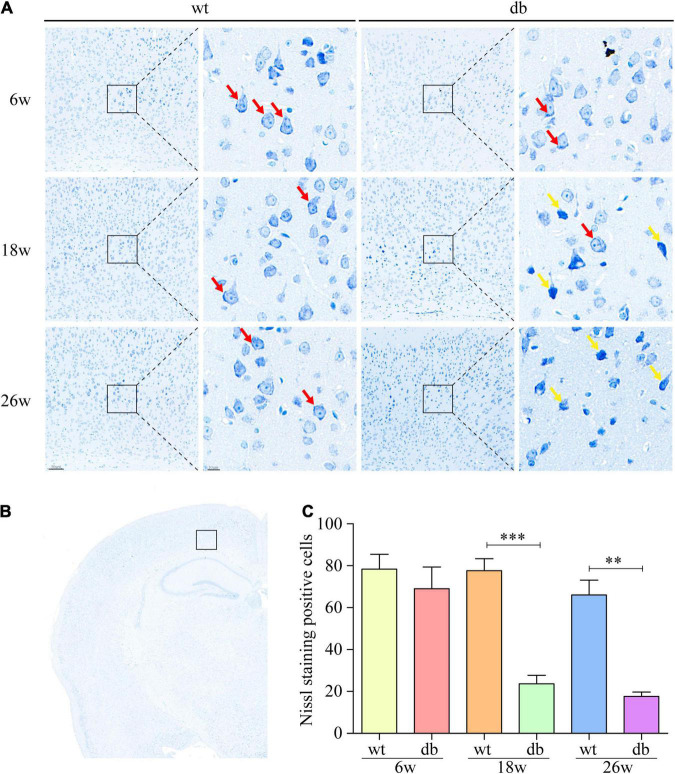
Nissl staining was used to analyze the pathological changes of neuronal cells in mice. **(A,B)** Representative Nissl staining of neurons in the cortex at different ages. Scale bar = 50 or 10 μm. The red arrow points to the Nissl body. The yellow arrow points to damaged neurons. **(C)** Quantitative analysis of Nissl body positive cells. (*n* = 3 per group); ^**^P < 0.01; ^***^P < 0.001.

Subsequently, Nissl staining was performed to assess the pathological morphology of neuronal cells in mouse brain. Nissl body is an indicator that reflects the functional state of neurons. When neurons are damaged, the Nissl bodies would decrease or even disappear. We found that the neurons exhibited a normal morphology with distinct nuclei and abundant Nissl bodies in the cytoplasm in the WT mice of all ages (Figures 2A–C). On the other hand, the neurons randomly showed nuclear condensation, sparse Nissl bodies and abnormal staining at 18 and 26 weeks of age in db/db mice (Figures 2A–C).

**FIGURE 3:**
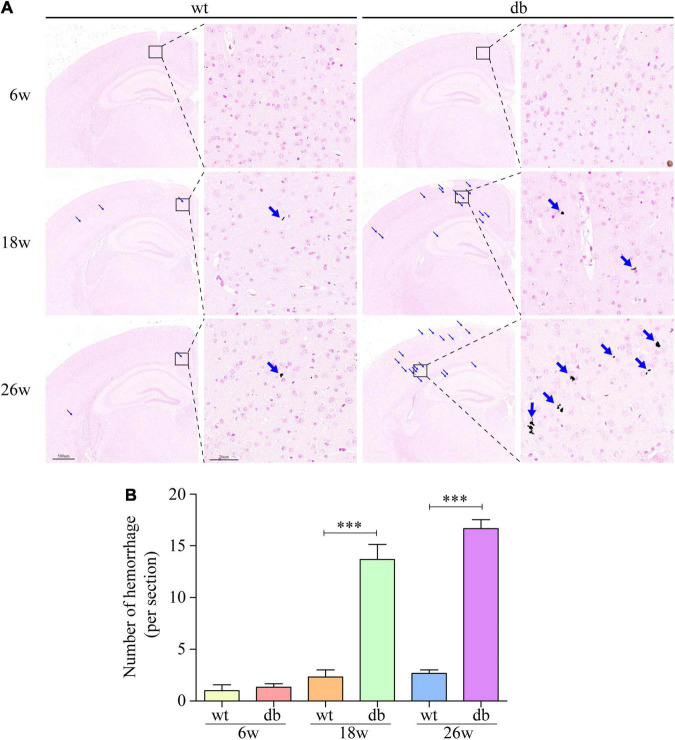
Prussian blue staining was used to detect the microhemorrhage in mice. **(A)** Representative images of hemorrhages in the cortex. Scale bar = 500 or 20 μm. The red arrow points to the hemorrhages. **(B)** Quantitative analysis of hemorrhages. (*n* = 3 per group); ^***^P < 0.001.

It is reported that microhemorrhage is a common pathological feature of db/db mice. Therefore, Prussian blue staining was used to further detect the microhemorrhage in the brain tissue of each group of mice. The results revealed that only negligible microhemorrhages could be detected in 6-week-old db/db and WT mice. However, significant increase number of microhemorrhages were observed in db/db mice at the age of 18 and 26 weeks when compared to age-matched WT mice (Figures 3A,B). Taken together, these results indicate that the pathological changes of brain tissues aggravate during the progression of T2DM in db/db mice.

### Neuroinflammation Was Prominent in Db/db Mice

**FIGURE 4:**
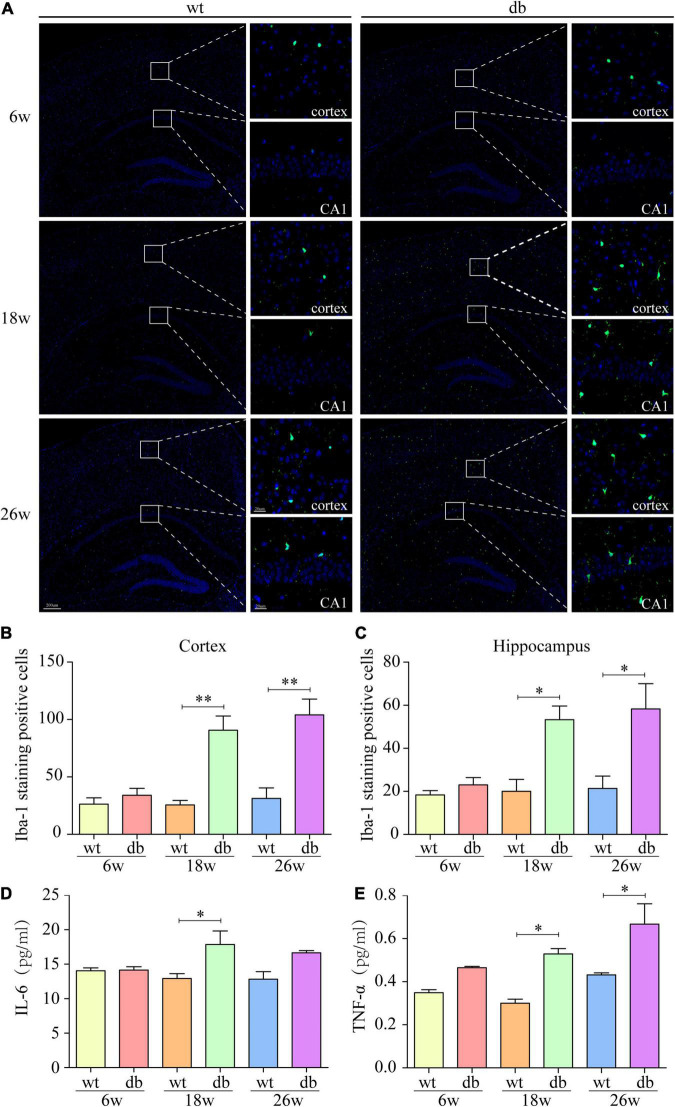
Increased microgliosis and pro-inflammatory cytokines in db/db mice with age. **(A)** Representative immunofluorescent staining of Iba-1-positive microglia in the cortex and hippocampus of mice. Scale bar = 200 or 20 μm. **(B,C)** Quantification of Iba-1-staining positive cells within the cortex or the hippocampus. **(D,E)** MSD was performed to detect the protein levels of IL-6 and TNF-α. (*n* = 3 per group); *P < 0.05; ^**^P < 0.01.

To evaluate the role of neuroinflammation on T2DM-mediated cognitive dysfunction and neuron injury, microglial activation was assessed by quantifying Iba-1 immunoreactivity and the production of pro-inflammatory cytokines, including IL-6 and TNF-α. Analysis showed that Iba-1 positive cells in the hippocampus and cortex were not significantly different between db/db and WT mice at 6 weeks of age. At 18 and 26 weeks of age, db/db mice displayed a significant increase of activated microglia in both the hippocampus and cortex compared to the age-matched WT mice (Figures 4A–C). We further evaluated the expression of pro-inflammatory cytokines including IL-6 and TNF-α in the whole brain. Analysis by MSD revealed that IL-6 and TNF-α expressions remained unaffected in both WT and db/db mice at 6 weeks of age, whereas a dramatically increase of IL-6 and TNF-α expressions were observed in 18-week-old db/db mice compared with WT mice. At 26 weeks of age, when compared with WT mice, the expression of TNF-α in db/db mice was significantly increased, and the trend of IL-6 expression was consistent with TNF-α but without significant statistical difference (Figures 4D,E). The results demonstrate that T2DM could promote microglia activation and subsequent production of pro-inflammatory cytokines in db/db mice with age.

### Alterations in Gut Microbiome Composition in Db/db Mice

**FIGURE 5:**
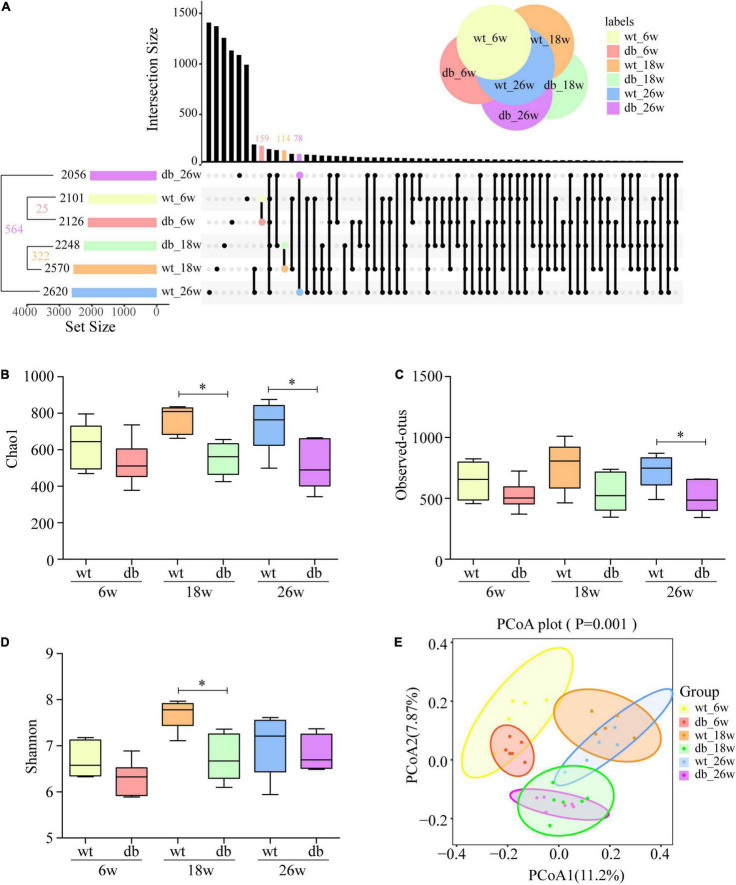
Alpha and beta diversities of the gut microbiota in db/db mice with age. **(A)** UpSet plot for the quantitative analysis of sets and intersections in each group. **(B–D)** Variation in alpha diversity (Chao1, Observed-otus, and Shannon) in mice at different ages. **(E)** PCoA analysis of gut microbiota. (*n* = 6 per group); *P < 0.05.

To delineate the influence of T2DM and age on the diversity of gut microbiota, we use the UpSet plot, an advanced Venn diagram, for the quantitative analysis of sets and their intersections (Ballarini et al., 2020). The analysis showed that the number of gut microbiota increased with age in both WT and db/db mice, whereas WT mice increased significantly compared with age-matched db/db mice. Intriguingly, the intersection of db/db mice and age-matched WT mice decreased with age (Figure 5A). To further determine the differences of gut microbiota diversity, the alpha and beta diversities were evaluated. The within-sample alpha diversity analysis showed a significant decrease in gut microbial community evenness and richness of 18- and 26-week-old db/db mice compared with age-matched WT mice based on Chao 1, Observed-otus, and Shannon indices. The alpha diversity showed a slight decrease in db/db mice than in WT mice at 6 weeks of age, but the result was not significant difference (Figures 5B–D). Principal coordinate analysis (PCoA) based on unweighted Unifrac was used to measure beta diversity and visualize the bacterial composition dissimilarity among each group. The analysis showed that the cluster for db/db mice was similar to WT mice at the age of 6 weeks. Nevertheless, the clusters for db/db mice were clear separated from WT mice at 18 and 26 weeks of age (Figure 5E). Overall, these results suggest that both T2DM and age have an effect on the gut microbiota composition of db/db mice.

### Differentially Represented Bacterial Taxa in Db/db Mice

**FIGURE 6:**
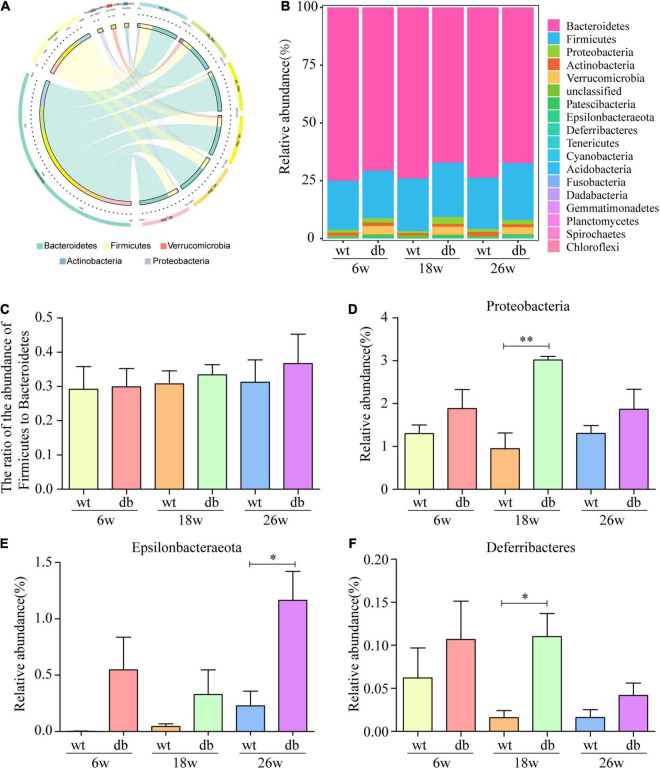
Alteration of gut microbiota composition in db/db mice at the phylum level. **(A,B)** The Circos and bar chart of common bacterial communities in each group. **(C)** The ratio of the abundance of Firmicutes to Bacteroidetes in each group. **(D–F)** The relative abundance of three bacterial communities in each group. (*n* = 6 per group); *P < 0.05; ^**^P < 0.01.

**FIGURE 7:**
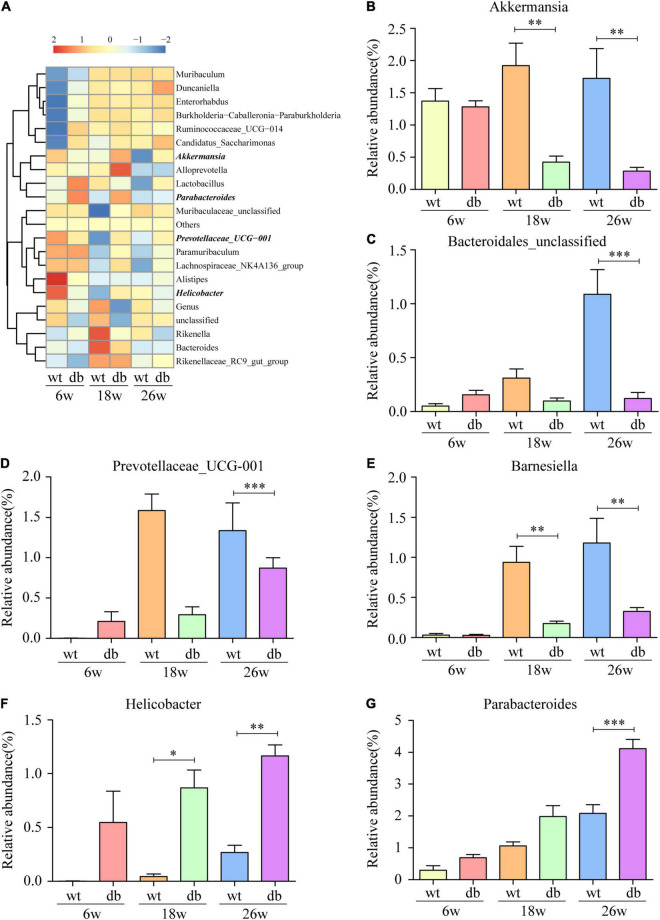
Alteration of gut microbiota composition in db/db mice at the genus level. **(A)** Community heatmap at the genus level. **(B–G)** The relative abundance of six bacterial communities in each group. (*n* = 6 per group); *P < 0.05; ^**^P < 0.01; ^***^P < 0.001.

The analysis of the gut microbiota composition at the phylum and genus levels showed specific differences between the WT and db/db mice at different ages. Among the common bacterial communities, Bacteroidetes, Firmicutes, Proteobacteria, Actinobacteria, and Verrucomicrobia were the main five phyla present in the gut microbiota of all groups (Figures 6A,B). Many studies have shown that obesity and diabetes mellitus are closely related to the ratio of Firmicutes to Bacteroidetes (Ley et al., 2005; Grigor’eva, 2020; Hung et al., 2021). However, the results revealed a slight increase of the Firmicutes/Bacteroidetes ratio in db/db mice than in WT mice at each age, but without significant difference (Figure 6C). In addition, no significant differences were observed in microbiota composition between the 6-week-old db/db and WT mice at the phylum or genus level (Figures 6, 7). Compared to age-matched WT mice, the relative abundance of phylum Proteobacteria, phylum Deferribacteres, and genus Helicobacter were significant higher in db/db mice at 18 weeks, whereas the relative abundance of genus Akkermansia and genus Barnesiella were significant lower in db/db mice at 18 weeks (Figures 6D,F, 7B,E). Moreover, compared to age-matched WT mice, the relative abundance of phylum Epsilonbacteraeota, genus Helicobacter, and genus Parabacteroides increased significantly in db/db mice at 26 weeks, whereas the relative abundance of genus Akkermansia, genus Barnesiella, genus Bacteroidales-unclassified, and genus Prevotellaceae-UCG-001 decreased significantly in db/db mice at 26 weeks (Figures 6E, 7B–G). The results above collectively indicate that the abundance of several gut bacteria differ significantly between the WT and db/db mice as T2DM progresses.

### Correlation Analysis of Mouse Phenotypes and Gut Bacteria

**FIGURE 8:**
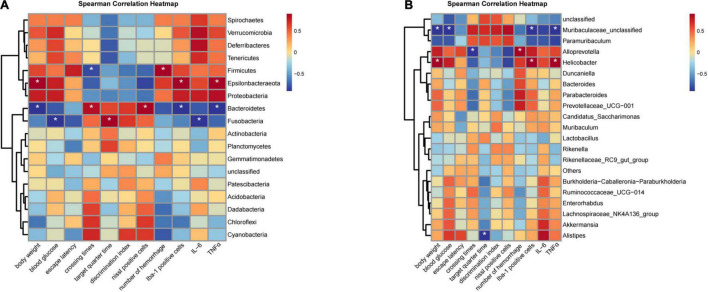
Heatmaps showing correlations between phenotypes and gut microbiota. **(A)** Correlation analysis at the phylum level. **(B)** Correlation analysis at the genus level. (*n* = 6 per group); *P < 0.05.

Spearman correlation analysis was used to evaluate the significant difference between mouse phenotypes and phylum/genus-level gut bacteria. At the phylum level, the relative abundance of Bacteroidetes was positively associated with crossing times and Nissl staining positive cells while negatively correlated with body weight, Iba-1 positive cells, and the expression of TNF-α (Figure 8A). Firmicutes was positively correlated with the number of hemorrhage while negatively correlated with crossing times (Figure 8A). The change in Epsilonbacteraeota levels was positively associated with body weight, Iba-1 positive cells, and the expression of TNF-α (Figure 8A). At the genus level, correlation analysis revealed a positive association between the relative abundance of Helicobacter and body weight, Iba-1 positive cells, and the expression of TNF-α (Figure 8B). Muribaculaceae-unclassified was negatively correlated with body weight, blood glucose, Iba-1 positive cells, and the expression of TNF-α (Figure 8B).

## Discussion

Recent studies have shown possible links between gut microbiota and T2DM-related cognitive dysfunction (Yu et al., 2019; Zhang Y. et al., 2021). However, there is no report on the relationship between gut microbiota and cognitive dysfunction during the progression of T2DM. Therefore, we investigated the longitudinal changes of gut microbiota and cognition in db/db mice from an early age, using 16S ribosomal RNA sequencing combined with conventional behavioral tests.

In the present study, we used db/db mice, a typical T2DM mouse model, to characterize the natural progression of the metabolic disorder and its effect on pathological changes of the central nervous system, especially cognitive function. Similar to previous reports, we observed that db/db mice exhibited obesity and hyperglycemia phenotypes with age, indicating that a stable T2DM phenotype was formed. It has been reported that aged db/db mice have an increased risk of spontaneous hemorrhage compared with mice without diabetes and are closely related to cognitive deficits (Ramos-Rodriguez et al., 2013). Consistently, our study also found that spontaneous hemorrhage became more pronounced as diabetes progressed in db/db mice. The above data are in accordance with clinical studies that prolonged hyperglycemia is associated with microvascular complications (Kruyt et al., 2010; Luitse et al., 2012). Evidence shows that diabetes is closely related to cognitive dysfunction, and both the level of hyperglycemia and the duration of diabetes are associated with cognitive dysfunction (Rawlings et al., 2019). Recent epidemiological studies have found that compared with non-diabetic individuals, diabetic patients have an increased risk of cognitive dysfunction and even dementia (Huang et al., 2014). The MWM is widely used to assess spatial learning and memory. Previous studies have reported that the cognitive function of diabetic mice is impaired, and it worsens with the prolongation of diabetes (Ramos-Rodriguez et al., 2013). Similar to the previous study, our study found that the pre-diabetic mice (6 weeks of age) performed well at the MWM, but when the db/db mice reached 18 weeks of age, obvious cognitive dysfunction appeared, showing inferior learning and memory performance. Since the swimming speed of db/db mice at 18 and 26 weeks was significantly slower than that of the WT group, it may be considered that the behavioral results were biased due to the high body weight of these mice. However, the swimming speed of obese db/db mice at 6 weeks has already decreased, while spatial learning and memory are almost unaffected, indicating the synergistic effect of diabetes and age. NOR is a test less affected by motor ability (Ennaceur and Delacour, 1988). We further applied NOR analysis and confirmed the reliability of MWM, indicating that learning and memory capacities decrease with the progression of T2DM.

Neuroinflammation, including activation of glial cells and secretion of inflammatory cytokines, is increasingly recognized as the underlying pathogenesis of diabetes-associated cognitive dysfunction (Marioni et al., 2010; Dinel et al., 2011). Iba-1 is widely used as a specific marker of microglia. In the present study, we found that compared with WT mice, the number of Iba-1-positive cells in db/db mice showed a significant increase in the cortex and hippocampus as diabetes progressed, which is in consistent with the previous work where they showed that diabetes-induced cognitive dysfunction was accompanied by microgliosis (Infante-Garcia et al., 2017). It has also been reported that the levels of pro-inflammatory factors IL-6 and TNF-α are significantly increased in both STZ-induced diabetic rats and db/db mice (Miao et al., 2015; Lee and Yang, 2019). Consistently, our study found that the levels of IL-6 and TNF-α in db/db mice at 18 and 26 weeks were significantly higher than those in age-matched WT mice.

The gut microbiota, composed of trillions of symbiotic microorganisms, is essential for host’s health and survival (Fung et al., 2017). In addition, microbial diversity usually increases after birth, and microbial composition changes gradually during late childhood, adolescence, and adulthood (Odamaki et al., 2016; Kundu et al., 2017; Chen et al., 2020). We observed a trend toward increased diversity and richness from 6 to 26 weeks in both WT and db/db mice as indicated by the UpSet plot, Chao 1, Observed-otus, and Shannon indices, suggesting aging is an important factor affecting microbial composition. Moreover, the gut microbiota has been implicated to play an important role in regulating immunity and metabolism (Petra et al., 2015; Ochoa-Reparaz and Kasper, 2016) and in the development of obesity and T2DM (Ten Kulve et al., 2015, 2016; Wijdeveld and Nieuwdorp, 2020). It has been reported that short-chain fatty acids derived from the metabolites of Akkermansia muciniphila can bind to a few G protein-coupled receptors to activate signaling pathways and participate in the regulation of blood glucose levels (Zhai et al., 2019). Previous studies have also shown that abnormal blood glucose levels were related to changes in gut microbiota (Tilg and Moschen, 2014; Wen and Duffy, 2017). The increase in blood glucose has been reported to be accompanied with an decrease in the relative abundance of Bacteroides acidifaciens, Butyricimonas virosa, Bacteroides eggerthii, and Desulfovibrio oxamicus in the gut (Zheng et al., 2021). From PCoA results, we found that the clusters of db/db mice at 18 and 26 weeks were significantly separated from the clusters of age-matched WT mice, indicating that the gut composition changes as T2DM progresses. Furthermore, we observed that the differences in bacterial taxa between the WT and db/db mice increased with age, which coincides with the progression of cognitive dysfunction and microglial activation in db/db mice. Growing evidence reports that the gut microbiota plays a preeminent role in the pathogenesis of central nervous system diseases via the gut–brain axis (Mayer, 2011). Previous study has demonstrated that the gut microbiota composition and relative abundance were strongly associated with cognitive dysfunction in STZ-induced diabetic mice (Yu et al., 2019). We found that compared to age-matched WT mice, the relative abundance of phylum Proteobacteria, phylum Deferribacteres, and genus Helicobacter were significant higher in db/db mice at 18 weeks, whereas the relative abundance of genus Akkermansia and genus Barnesiella were significant lower in db/db mice at 18 weeks. Akkermansia muciniphila, a mucin-degrading bacteria that resides in the mucus layer, is considered as a promising probiotic candidate (Collado et al., 2007). It is reported that the abundance of Akkermansia inversely correlates with obesity and T2DM in mice and humans (Derrien et al., 2004; Belzer and de Vos, 2012; Everard et al., 2013). Recent studies have also shown that Akkermansia treatment reduces hippocampal microgliosis and proinflammatory cytokines by improving gut permeability, ultimately alleviating cognitive dysfunction of high-fat diet-fed mice (Yang et al., 2019). Altered Proteobacteria and Helicobacter composition has been reported to be implicated in inflammatory reactions and cognitive function (Kountouras et al., 2009; Shin et al., 2015; Berrett et al., 2016; Vogt et al., 2017). Similarly, we found that the increase in the relative abundance of Helicobacter is positively associated with body weight, Iba-1 positive cells, and the expression of TNF-α, suggesting that Helicobacter may be a sensitive indicator of neuroinflammation in db/db mice. We also observed that the number of crossing platforms is positively correlated with Bacteroidetes and negatively correlated with Firmicutes. These data provide potential non-invasive biomarkers for the diagnosis of diabetic cognitive dysfunction.

Overall, the current longitudinal research suggests that abnormal gut microbiota composition may contribute to diabetic cognitive impairment by regulating neuroinflammation. Nevertheless, future work is warranted to determine the underlying molecular mechanism of the gut microbiota on the diabetes-related cognitive dysfunction.

## Data Availability Statement

The datasets presented in this study can be found in online repositories. The name of the repository and accession number can be found below: National Center for Biotechnology (NCBI) BioProject (https://www.ncbi.nlm.nih.gov/bioproject/, PRJNA784719).

## Ethics Statement

The animal study was reviewed and approved by the Ethical Committee on Animal Welfare of Shanghai Jiao Tong University Affiliated Sixth People’s Hospital.

## Author Contributions

JZ and XW designed the study and contributed to the production of the manuscript. XW and YuZ supervised the research. JZ, YaZ, and YY performed the experiments. JZ and LL analyzed the data. All authors read and approved the final manuscript.

## Conflict of Interest

The authors declare that the research was conducted in the absence of any commercial or financial relationships that could be construed as a potential conflict of interest.

## Publisher’s Note

All claims expressed in this article are solely those of the authors and do not necessarily represent those of their affiliated organizations, or those of the publisher, the editors and the reviewers. Any product that may be evaluated in this article, or claim that may be made by its manufacturer, is not guaranteed or endorsed by the publisher.

## References

[B1] Bakir-Gungor B. Bulut O. Jabeer A. Nalbantoglu O. U. Yousef M. (2021). Discovering Potential Taxonomic Biomarkers of Type 2 Diabetes From Human Gut Microbiota via Different Feature Selection Methods. *Front. Microbiol.* 12:628426. 10.3389/fmicb.2021.628426 34512559 PMC8424122

[B2] Ballarini N. M. Chiu Y. D. Konig F. Posch M. Jaki T. (2020). A critical review of graphics for subgroup analyses in clinical trials. *Pharm. Stat.* 19 541–560. 10.1002/pst.2012 32216035 PMC8647927

[B3] Belzer C. de Vos W. M. (2012). Microbes inside–from diversity to function: the case of Akkermansia. *ISME J.* 6 1449–1458. 10.1038/ismej.2012.6 22437156 PMC3401025

[B4] Berrett A. N. Gale S. D. Erickson L. D. Brown B. L. Hedges D. W. (2016). Folate and Inflammatory Markers Moderate the Association Between *Helicobacter* pylori Exposure and Cognitive Function in US Adults. *Helicobacter* 21 471–480. 10.1111/hel.12303 26935014

[B5] Cattaneo A. Cattane N. Galluzzi S. Provasi S. Lopizzo N. Festari C. (2017). Association of brain amyloidosis with pro-inflammatory gut bacterial taxa and peripheral inflammation markers in cognitively impaired elderly. *Neurobiol. Aging* 49 60–68. 10.1016/j.neurobiolaging.2016.08.019 27776263

[B6] Chen Y. Fang L. Chen S. Zhou H. Fan Y. Lin L. (2020). Gut Microbiome Alterations Precede Cerebral Amyloidosis and Microglial Pathology in a Mouse Model of Alzheimer’s Disease. *Biomed. Res. Int.* 2020:8456596. 10.1155/2020/8456596 32596386 PMC7273394

[B7] Collado M. C. Derrien M. Isolauri E. de Vos W. M. Salminen S. (2007). Intestinal integrity and Akkermansia muciniphila, a mucin-degrading member of the intestinal microbiota present in infants, adults, and the elderly. *Appl. Environ. Microbiol.* 73 7767–7770. 10.1128/AEM.01477-07 17933936 PMC2168041

[B8] Collins S. M. Surette M. Bercik P. (2012). The interplay between the intestinal microbiota and the brain. *Nat. Rev. Microbiol.* 10 735–742. 23000955 10.1038/nrmicro2876

[B9] Derrien M. Vaughan E. E. Plugge C. M. de Vos W. M. (2004). Akkermansia muciniphila gen. nov., sp. nov., a human intestinal mucin-degrading bacterium. *Int. J. Syst. Evol. Microbiol.* 54 1469–1476. 10.1099/ijs.0.02873-0 15388697

[B10] Dinel A. L. Andre C. Aubert A. Ferreira G. Laye S. Castanon N. (2011). Cognitive and emotional alterations are related to hippocampal inflammation in a mouse model of metabolic syndrome. *PLoS One* 6:e24325. 10.1371/journal.pone.0024325 21949705 PMC3174932

[B11] Ennaceur A. Delacour J. (1988). A new one-trial test for neurobiological studies of memory in rats. 1: Behavioral data. *Behav. Brain Res.* 31 47–59. 10.1016/0166-4328(88)90157-x 3228475

[B12] Everard A. Belzer C. Geurts L. Ouwerkerk J. P. Druart C. Bindels L. B. (2013). Cross-talk between Akkermansia muciniphila and intestinal epithelium controls diet-induced obesity. *Proc. Natl. Acad. Sci. U S A.* 110 9066–9071. 10.1073/pnas.1219451110 23671105 PMC3670398

[B13] Fung T. C. Olson C. A. Hsiao E. Y. (2017). Interactions between the microbiota, immune and nervous systems in health and disease. *Nat. Neurosci.* 20 145–155. 10.1038/nn.4476 28092661 PMC6960010

[B14] Grigor’eva I. N. (2020). Gallstone Disease, Obesity and the Firmicutes/Bacteroidetes Ratio as a Possible Biomarker of Gut Dysbiosis. *J. Pers. Med.* 11:13. 10.3390/jpm11010013 33375615 PMC7823692

[B15] Huang C. C. Chung C. M. Leu H. B. Lin L. Y. Chiu C. C. Hsu C. Y. (2014). Diabetes mellitus and the risk of Alzheimer’s disease: a nationwide population-based study. *PLoS One* 9:e87095. 10.1371/journal.pone.0087095 24489845 PMC3906115

[B16] Hung W. C. Hung W. W. Tsai H. J. Chang C. C. Chiu Y. W. Hwang S. J. (2021). The Association of Targeted Gut Microbiota with Body Composition in Type 2 Diabetes Mellitus. *Int. J. Med. Sci.* 18 511–519. 10.7150/ijms.51164 33390820 PMC7757146

[B17] Infante-Garcia C. Jose Ramos-Rodriguez J. Marin-Zambrana Y. Teresa Fernandez-Ponce M. Casas L. Mantell C. (2017). Mango leaf extract improves central pathology and cognitive impairment in a type 2 diabetes mouse model. *Brain Pathol.* 27 499–507. 10.1111/bpa.12433 27537110 PMC8029052

[B18] Jackson L. Dumanli S. Johnson M. H. Fagan S. C. Ergul A. (2020). Microglia knockdown reduces inflammation and preserves cognition in diabetic animals after experimental stroke. *J. Neuroinflamm.* 17:137. 10.1186/s12974-020-01815-3 32345303 PMC7189436

[B19] Karlsson F. H. Tremaroli V. Nookaew I. Bergstrom G. Behre C. J. Fagerberg B. (2013). Gut metagenome in European women with normal, impaired and diabetic glucose control. *Nature* 498 99–103. 10.1038/nature12198 23719380

[B20] Kountouras J. Boziki M. Gavalas E. Zavos C. Grigoriadis N. Deretzi G. (2009). Eradication of *Helicobacter* pylori may be beneficial in the management of Alzheimer’s disease. *J. Neurol.* 256 758–767. 19240960 10.1007/s00415-009-5011-z

[B21] Kruyt N. D. Biessels G. J. DeVries J. H. Luitse M. J. Vermeulen M. Rinkel G. J. (2010). Hyperglycemia in aneurysmal subarachnoid hemorrhage: a potentially modifiable risk factor for poor outcome. *J. Cereb. Blood Flow Metab.* 30 1577–1587. 10.1038/jcbfm.2010.102 20628402 PMC2949259

[B22] Kundu P. Blacher E. Elinav E. Pettersson S. (2017). Our Gut Microbiome: The Evolving Inner Self. *Cell* 171 1481–1493. 10.1016/j.cell.2017.11.024 29245010

[B23] Lee H. J. Yang S. J. (2019). Supplementation with Nicotinamide Riboside Reduces Brain Inflammation and Improves Cognitive Function in Diabetic Mice. *Int. J. Mol. Sci.* 20:4196. 10.3390/ijms20174196 31461911 PMC6747453

[B24] Ley R. E. Backhed F. Turnbaugh P. Lozupone C. A. Knight R. D. Gordon J. I. (2005). Obesity alters gut microbial ecology. *Proc. Natl. Acad. Sci. U S A.* 102 11070–11075. 10.1073/pnas.0504978102 16033867 PMC1176910

[B25] Liu T. Lee J. E. Wang J. Ge S. Li C. (2020). Cognitive Dysfunction in Persons with Type 2 Diabetes Mellitus: A Concept Analysis. *Clin. Nurs. Res.* 29 339–351. 10.1177/1054773819862973 31353950

[B26] Luitse M. J. Biessels G. J. Rutten G. E. Kappelle L. J. (2012). Diabetes, hyperglycaemia, and acute ischaemic stroke. *Lancet Neurol.* 11 261–271. 10.1016/s1474-4422(12)70005-4 22341034

[B27] Marioni R. E. Strachan M. W. Reynolds R. M. Lowe G. D. Mitchell R. J. Fowkes F. G. (2010). Association between raised inflammatory markers and cognitive decline in elderly people with type 2 diabetes: the Edinburgh Type 2 Diabetes Study. *Diabetes* 59 710–713. 10.2337/db09-1163 19959761 PMC2828661

[B28] Mayer E. A. (2011). Gut feelings: the emerging biology of gut-brain communication. *Nat. Rev. Neurosci.* 12 453–466. 10.1038/nrn3071 21750565 PMC3845678

[B29] Miao Y. He T. Zhu Y. Li W. Wang B. Zhong Y. (2015). Activation of Hippocampal CREB by Rolipram Partially Recovers Balance Between TNF-alpha and IL-10 Levels and Improves Cognitive Deficits in Diabetic Rats. *Cell Mol. Neurobiol.* 35 1157–1164. 10.1007/s10571-015-0209-3 26001770 PMC11488058

[B30] Ochoa-Reparaz J. Kasper L. H. (2016). The Second Brain: Is the Gut Microbiota a Link Between Obesity and Central Nervous System Disorders? *Curr. Obes. Rep.* 5 51–64. 10.1007/s13679-016-0191-1 26865085 PMC4798912

[B31] Odamaki T. Kato K. Sugahara H. Hashikura N. Takahashi S. Xiao J. Z. (2016). Age-related changes in gut microbiota composition from newborn to centenarian: a cross-sectional study. *BMC Microbiol.* 16:90. 10.1186/s12866-016-0708-5 27220822 PMC4879732

[B32] Petra A. I. Panagiotidou S. Hatziagelaki E. Stewart J. M. Conti P. Theoharides T. C. (2015). Gut-Microbiota-Brain Axis and Its Effect on Neuropsychiatric Disorders With Suspected Immune Dysregulation. *Clin. Ther.* 37 984–995. 10.1016/j.clinthera.2015.04.002 26046241 PMC4458706

[B33] Ramos-Rodriguez J. J. Ortiz O. Jimenez-Palomares M. Kay K. R. Berrocoso E. Murillo-Carretero M. I. (2013). Differential central pathology and cognitive impairment in pre-diabetic and diabetic mice. *Psychoneuroendocrinology* 38 2462–2475. 10.1016/j.psyneuen.2013.05.010 23790682

[B34] Rawlings A. M. Sharrett A. R. Albert M. S. Coresh J. Windham B. G. Power M. C. (2019). The Association of Late-Life Diabetes Status and Hyperglycemia With Incident Mild Cognitive Impairment and Dementia: The ARIC Study. *Diab. Care* 42 1248–1254. 10.2337/dc19-0120 31221696 PMC6609963

[B35] Sankar S. B. Infante-Garcia C. Weinstock L. D. Ramos-Rodriguez J. J. Hierro-Bujalance C. Fernandez-Ponce C. (2020). Amyloid beta and diabetic pathology cooperatively stimulate cytokine expression in an Alzheimer’s mouse model. *J. Neuroinflamm.* 17:38. 10.1186/s12974-020-1707-x 31992349 PMC6988295

[B36] Shin N. R. Whon T. W. Bae J. W. (2015). *Proteobacteria*: microbial signature of dysbiosis in gut microbiota. *Trends Biotechnol.* 33 496–503. 10.1016/j.tibtech.2015.06.011 26210164

[B37] Sun M. Ma K. Wen J. Wang G. Zhang C. Li Q. (2020). A Review of the Brain-Gut-Microbiome Axis and the Potential Role of Microbiota in Alzheimer’s Disease. *J. Alzheimers Dis.* 73 849–865. 10.3233/JAD-190872 31884474

[B38] Ten Kulve J. S. Veltman D. J. van Bloemendaal L. Barkhof F. Deacon C. F. Holst J. J. (2015). Endogenous GLP-1 mediates postprandial reductions in activation in central reward and satiety areas in patients with type 2 diabetes. *Diabetologia* 58 2688–2698. 10.1007/s00125-015-3754-x 26385462 PMC4630252

[B39] Ten Kulve J. S. Veltman D. J. van Bloemendaal L. Barkhof F. Drent M. L. Diamant M. (2016). Liraglutide Reduces CNS Activation in Response to Visual Food Cues Only After Short-term Treatment in Patients With Type 2 Diabetes. *Diab. Care* 39 214–221. 10.2337/dc15-0772 26283736

[B40] Tilg H. Moschen A. R. (2014). Microbiota and diabetes: an evolving relationship. *Gut* 63 1513–1521. 10.1136/gutjnl-2014-306928 24833634

[B41] Umegaki H. (2018). Diabetes-related cognitive dysfunction: Hyperglycemia in the early stage might be a key? *J. Diab. Investig.* 9 1019–1021. 10.1111/jdi.12808 29377593 PMC6123037

[B42] van Sloten T. T. Sedaghat S. Carnethon M. R. Launer L. J. Stehouwer C. D. A. (2020). Cerebral microvascular complications of type 2 diabetes: stroke, cognitive dysfunction, and depression. *Lancet Diabetes Endocrinol.* 8 325–336. 10.1016/S2213-8587(19)30405-X 32135131 PMC11044807

[B43] Villarreal S. Zhao F. Hyde L. A. Holder D. Forest T. Sondey M. (2017). Chronic Verubecestat Treatment Suppresses Amyloid Accumulation in Advanced Aged Tg2576-AbetaPPswe Mice Without Inducing Microhemorrhage. *J. Alzheimers Dis.* 59 1393–1413. 10.3233/JAD-170056 28800329 PMC5611839

[B44] Vogt N. M. Kerby R. L. Dill-McFarland K. A. Harding S. J. Merluzzi A. P. Johnson S. C. (2017). Gut microbiome alterations in Alzheimer’s disease. *Sci. Rep.* 7:13537. 29051531 10.1038/s41598-017-13601-y PMC5648830

[B45] Wang H. Y. Wu M. Diao J. L. Li J. B. Sun Y. X. Xiao X. Q. (2020). Huperzine A ameliorates obesity-related cognitive performance impairments involving neuronal insulin signaling pathway in mice. *Acta Pharmacol. Sin.* 41 145–153. 10.1038/s41401-019-0257-1 31213670 PMC7471460

[B46] Wen L. Duffy A. (2017). Factors Influencing the Gut Microbiota, Inflammation, and Type 2 Diabetes. *J. Nutr.* 147 1468S–1475S. 10.3945/jn.116.240754 28615382 PMC5483960

[B47] Wijdeveld M. Nieuwdorp M. (2020). R IJ: The interaction between microbiome and host central nervous system: the gut-brain axis as a potential new therapeutic target in the treatment of obesity and cardiometabolic disease. *Expert Opin. Ther. Targets* 24 639–653. 10.1080/14728222.2020.1761958 32441559

[B48] Yang Y. Zhong Z. Wang B. Xia X. Yao W. Huang L. (2019). Early-life high-fat diet-induced obesity programs hippocampal development and cognitive functions via regulation of gut commensal Akkermansia muciniphila. *Neuropsychopharmacology* 44 2054–2064. 10.1038/s41386-019-0437-1 31207607 PMC6897910

[B49] Yu F. Han W. Zhan G. Li S. Xiang S. Zhu B. (2019). Abnormal gut microbiota composition contributes to cognitive dysfunction in streptozotocin-induced diabetic mice. *Aging* 11 3262–3279. 10.18632/aging.101978 31123221 PMC6555457

[B50] Zhai Q. Feng S. Arjan N. Chen W. (2019). A next generation probiotic, Akkermansia muciniphila. *Crit. Rev. Food Sci. Nutr.* 59 3227–3236. 30373382 10.1080/10408398.2018.1517725

[B51] Zhang J. Zheng Y. Zhao Y. Zhang Y. Liu Y. Ma F. (2021). Andrographolide ameliorates neuroinflammation in APP/PS1 transgenic mice. *Int. Immunopharmacol.* 96:107808. 10.1016/j.intimp.2021.107808 34162168

[B52] Zhang Y. Lu S. Yang Y. Wang Z. Wang B. Zhang B. (2021). The diversity of gut microbiota in type 2 diabetes with or without cognitive impairment. *Aging Clin. Exp. Res.* 33 589–601. 10.1007/s40520-020-01553-9 32301029

[B53] Zhao Q. Zhang F. Yu Z. Guo S. Liu N. Jiang Y. (2019). HDAC3 inhibition prevents blood-brain barrier permeability through Nrf2 activation in type 2 diabetes male mice. *J. Neuroinflamm.* 16:103. 10.1186/s12974-019-1495-3 31101061 PMC6525453

[B54] Zheng S. Wang Y. Fang J. Geng R. Li M. Zhao Y. (2021). Oleuropein Ameliorates Advanced Stage of Type 2 Diabetes in db/db Mice by Regulating Gut Microbiota. *Nutrients* 13:2131. 10.3390/nu13072131 34206641 PMC8308455

